# Dietary quality indices modifies the effects of melanocortin-4 receptor (MC4R) rs17782313 polymorphism on cardio-metabolic risk factors and hypothalamic hormones in obese adults

**DOI:** 10.1186/s12872-020-01366-8

**Published:** 2020-02-04

**Authors:** Mahdieh Khodarahmi, Houman Kahroba, Mohammad Asghari Jafarabadi, Mehran Mesgari-Abbasi, Mahdieh Abbasalizad Farhangi

**Affiliations:** 1 Student Research Committee, Department of Nutrition, Faculty of Nutrition and Food Science, Tabriz University of Medical Sciences, Tabriz, Iran; 2 Molecular Medicine Research Center, Tabriz University of Medical Sciences, Tabriz, Iran; 3 Road Traffic Injury Research Center, Tabriz University of Medical Sciences, Tabriz, Iran; 4 Department of Statistics and Epidemiology, Faculty of Health, Tabriz University of Medical Sciences, Tabriz, Iran; 5 Nutrition Research Center, Tabriz University of Medical Sciences, Attar-neishabouri Ave, Golgasht St, Tabriz, 5165665931 Iran

**Keywords:** Diet quality, Obesity, Gene-diet interaction, MC4R, Cardio-metabolic risk

## Abstract

**Background:**

Although the Melanocortin-4 Receptor (MC4R) gene rs17782313 C/T has been consistently related to obesity risk, the interaction between MC4R polymorphism and diet quality indices on cardio-metabolic risk factors has not yet investigated. Therefore we aimed to test this hypothesis.

**Methods:**

This cross-sectional study recruited 188 (96 males and 92 females) healthy obese adults aged 20–50 years. Diet quality indices including Healthy Eating Index-2015 (HEI-2015) and Diet Quality Index-International (DQI-I) were constructed using data from a validated food frequency questionnaire. MC4R s17782313 were genotyped by Polymerase Chain Reaction-Restriction Fragment Length Polymorphism (PCR-RFLP). The interaction between MC4R polymorphism and diet quality indices was tested by Analysis of covariance (ANCOVA) multivariate interaction model.

**Results:**

There were significant gene-diet interactions between rs17782313 and HEI-2015 (P _Interaction_ < 0.05) in modulating low-density lipoprotein cholesterol (LDL-C) levels among female group; rare allele heterozygotes of rs17782313 had highest mean of LDL-C concentration when placed in second tertile of HEI (*P* < 0.05). Moreover, rs17782313 and both indices (HEI and DQI-I) had significant interaction on serum glucose concentrations, systolic and diastolic blood pressure (SBP, DBP) in males (P _Interaction_ < 0.05); when adherence to these indices was low, the obesity risk allele was associated with serum glucose concentrations, SBP and DBP. These gene-diet interactions remained significant even after adjustment for potential confounders.

**Conclusion:**

Our study showed that MC4R rs17782313 interacts with adherence to the dietary quality indices (HEI and DQI-I) to influence several cardio-metabolic risk factors in obese male and females. Further large prospective studies are warranted to confirm our findings.

## Background

Obesity, as a major public health concern, has increased prevalence worldwide [1, 2]. According to World Health Organization (WHO) report, over 650 million adults across the world are obese. Available data have also shown a risen rate of obesity among the Iranian population over the last three decades. Recently, it has been estimated that 21.7% of Iranian adults are obese [3]. A large body of evidence has shown that obesity is a strong risk factor for various chronic diseases including cardiovascular disease, diabetes, stroke and certain types of cancers [4]. Additionally, obesity, in all age groups, is related to more adverse cardio-metabolic risk factors, including hypertension, hypertriglyceridemia, and insulin resistance [5].

Since obesity is a chronic and complex condition, genetic and environmental factors, and their interaction play a critical role in prevention and development of obesity [6]. Diet, as a major lifestyle factor, plays a critical role in the prevention and development of obesity and its consequence non-communicable diseases (NCDs) [6, 7]. Traditional researches of diet and chronic diseases have usually focused on single foods and macro- and micronutrients without considering synergistic effect of highly correlated nutrients and interactions between food components [8]. Many investigators have suggested index-based dietary pattern approach as a strong and more informative tool for evaluating diet-disease associations [9].

Diet Quality Index-International (DQI-I), one of the predefined diet quality indexes, is developed to make comparisons of diet quality among countries experiencing different stages of the nutrition transition [10]. Although there are only few studies have assessed the association between DQI-I and obesity, the finding these researches are conflicting [11–13]. Additionally, Healthy Eating Index (HEI), as one of the most popular diet quality indicator in nutrition research, was constructed by United States Department of Agriculture to measure alignment with Dietary Guidelines for Americans (DGA) [14]. HEI-2015, the most recent updated version, reflects the 2015–2020 DGA by employing a density-based approach [15]. Prior versions of HEI have found to be inversely related to major chronic diseases such as cardiovascular disease, cancer, and Type 2 diabetes [16]. However, the results of previous studies regarding the association between this index and obesity are not consistent [11]. Moreover, there are scarce studies on the above association in developing countries [9]. On the other hand, the applicability of latest version of HEI (HEI-2015) in many chronic disease especially obesity has not yet assessed. Hence, it is required to examine efficacy of this new HEI score in developing nations.

Beside diet, genetic factors can also influence on susceptibility to obesity and its comorbidities. The melanocortin-4 receptor (MC4R), a plasma membrane G protein-coupled receptor, plays a central role in regulating appetite and energy homeostasis [17]. Activity of this receptor is regulated by endogenous neuropeptides including: alpha -melanocyte stimulating hormone (α-MSH) and agouti-related protein (AGRP) [18]. Previous evidence of animal studies has revealed that any defect and disruption in MC4R gene may be lead to obesity-related phenotypes [19]. A large-scale meta-analysis of seven genome-wide association studies (GWAS) in 2008 reported MC4R as the second association signal for common obesity [20]. Loos et al. [20] in this study found a single nucleotide polymorphism (SNP) near MC4R (rs17782313), located 188 kb downstream of the MC4R, which was strongly related to obesity in adults and children. Subsequently, the association between rs17782313-SNP and different obesity-related phenotypes has been investigated among populations with various ethnicities [21, 22]. However, some of the studies have found no association between rs17782313 and obesity-related cardio-metabolic traits [23–25]. This observed inconsistency might be due to the role of lifestyle factors specially diet in modulation of the effects of MC4R gene variation. On the other hand, scientific evidence has indicated that response to dietary modifications in treatment of obesity and its-related cardio-metabolic risk factors differs in various communities with different genetic structures [26]. So, it seems that interaction between diet and genetic susceptibility may explain this reciprocal effect, and these interactions may play an important role in development of obesity and its comorbidities [27]. So far, there is no study regarding the effect of the interaction between diet quality indices and rs17782313 polymorphism on cardio-metabolic risk factors and hypothalamic hormones (α-MSH and AgRP). Thus, the first aim of current study was to assess the association between genotypes of rs17782313 and cardio-metabolic risk factors and also diet quality indices (HEI-2015, DQI-I). Second, it was aimed to examine potential interactions between genotypes of rs17782313 and diet quality indices on cardio-metabolic risk factors and hypothalamic hormones in a group of obese Iranian population.

## Methods

### Subjects

The present research is a cross-sectional study carried out in Tabriz, Iran. Target population was 188 apparently healthy obese adults (92 males and 96 females) aged 20–50 years that were recruited using simple random sampling. The advertisements that provided general information about inclusion criteria (age 20 to 50 years, good health and obesity (body mass index (BMI) ≥ 30 kg/m^2^)) were used for recruiting eligible participants. Subjects were excluded if the following applied: pregnancy, lactation, and menopause, having any evidence of chronic disease such as hypertension, hyperlipidemia, cardio-vascular diseases, diabetes, hepatic disorders, renal disease, and cancer, or taking any medications effective for weight loss such as loop diuretics or corticosteroids, or antidepressants. Moreover, people taking lipid-lowering (e.g. statins) and antihypertensive medications were excluded. All protocols of this study were approved by Ethical Committee of the Tabriz University of Medical Sciences (registration code IR.TBZMED.REC.768). All of the subjects completed a written informed consent before participation in the study.

### Dietary assessment and calculation of dietary scores

Usual dietary intake was assessed by trained dietitians using a validated and reliable 147-item semi-quantitative food frequency questionnaire (FFQ) [28, 29]. Participants were asked to report frequency and amount of each food item consumed on a daily, weekly, monthly or yearly basis. Then, portion sizes of consumed foods were converted to gram using household measures [30]. Daily energy and nutrient intake were analyzed using Iranian Food Composition Table (FCT) [31] and complemented with the United States Department of Agriculture FCT [32].

### HEI-2015

HEI-2015, a measure of conformance to the 2015–2020 DGA, uses an energy density approach to scoring most components (i.e., scores per 1000 cal) [15]. This measure is made up of 13 components, 9 adequacy and 4 moderation components with a total score of 100 points. Total vegetables, total fruits (fruit, fruit juice and canned fruit), whole fruits (fruits except fruit juice), seafood and plant proteins, greens and beans, and total protein foods, scored 5 and 0 in the highest and lowest consumption, respectively. Other three adequacy components including whole grains, dairy and fatty acids were scored from 0 to 10, where 10 represents the highest consumption and 0 shows the lowest consumption. Moderation components include refined grains, sodium, added sugars, and saturated fats were scored in reverse (score 10 for the lowest consumption and score 0 for the highest consumption). It should be noted that the intermediate scores were computed proportionally. Higher total HEI-2015 scores reflecting a healthful diet and greater adherence to dietary guidelines.

### DQI-I

The Diet Quality Index – International (DQI-I) was constructed based on Kim et al’s method [10]. This indicator is constructed based on four categories of a high-quality diet including: variety, adequacy, moderation and overall balance. Total DQI-I score was computed by summing the scores of these categories, ranging from 0 to 100. There were sub-components for each of these categories. Dietary variety scores included diversity in food group and protein sources (20 points). Adequacy scores were adequate intake of fruits, vegetables, grains, fiber, protein, iron, vitamin C and calcium (40 points).

Moderation scores included total fat, saturated fat, cholesterol, sodium and empty calorie foods (30 points) and lastly overall balance score which capture balance of macronutrient and fatty acid ratios (10 points). A higher DQI-I score mean better diet quality.

### Demographic, anthropometric and blood pressure measures

Basic information including: age, gender, marital status, smoking and medical history were collected using standard questionnaires. Socioeconomic status (SES) was assessed through collecting information about occupational position, educational status, family size and house ownership as individual indicators. Then, total score was calculated. Information on Physical activity was evaluated using a self-administered short form of the International Physical Activity Questionnaire completed [33].

Body weight was measured with a Seca scale (Seca, Germany) to the nearest 100 g, while participants were minimally clothed without shoes. Height was also measured using a tape measure with a precision of 0.1 cm, in a standing position without shoes. Waist circumference (WC) was determined at narrowest area below the rib cage and above the umbilicus using a stretch-resistant tape measure to the nearest 0.1 cm over light clothing without applying any pressure to the body. Measurement of blood pressure was performed using a standardized mercury sphygmomanometer, after 15 min rest in a sitting position on chair. This measurement was conducted twice and the mean was considered as participant’s blood pressure.

### Mental health assessment

Self-administered the depression, Anxiety and Stress Scale 21 (DASS-21) questionnaire was employed for the mental health assessment component. The reliability (Cronbach’s alpha: 0.77, 0.79 and 0.78 for depression, anxiety and stress, respectively) and validity of this scale have previously been assessed among Iranian population [34, 35]. Each of the three scales, including depression, anxiety and stress has seven items and each answer is rated on a 4-point Likert scale, ranging from zero (“did not apply to me at all”) to 3 (“applied to me very much or most of the time”). A total score for each scale was determined by summing the relevant items scores. Based on the obtained overall score of each scale, subjects were classified into 5 categories: normal, mild, moderate, severe and extremely severe. Higher score of each three scale reflected greater degree of mood disruption.

### Appetite measurements

Appetite measurement was carried out using a 100-mm, visual analogue scale (VAS). VAS is a validated questionnaire and includes questions about feelings of hunger, satiation, fullness, prospective food consumption and the desire to eat something sweet, salty, or fat [36]. Subjects were requested to complete this questionnaire by making vertical mark on a 100 mm horizontal line for each question. VAS scores were calculated by measuring the distance from the left side of the line to the mark.

### Laboratory assessments

After an overnight fast, venous blood samples (10 ml) were taken from all subjects. These samples were centrifuged (10 min at 4500 rpm, 4 °C) to separate plasma and serum and then aliquoted and frozen at 80 C° until assay. Measurements of serum glucose, triglyceride (TG), total cholesterol and high-density lipoprotein cholesterol (HDL-C) were performed using a commercial kit (Pars Azmoon, Tehran, Iran).

Low-density lipoprotein cholesterol (LDL-C) concentration was calculated by the Friedewald equation [37]. Commercially available enzyme-linked immunosorbent assay (ELISA) kits (Bioassay Technology Laboratory, Shanghai Korean Biotech, Shanghai City, China) were used to detect plasma α-MSH and AgRP levels and also serum insulin concentration. The minimum level of detection of α-MSH and AgRP levels were 5.07 ng/L and 1.03 pg/ml, respectively. All these biochemical measurements were conducted according to the manufacturer’s protocol.

Homeostasis model assessment-insulin resistance index (HOMA-IR) and quantitative insulin sensitivity check index (QUICKI), the most used indices for evaluating insulin sensitivity, were calculated using standard formula [38, 39]. Atherogenic index of plasma (AIP) was calculated as Lg_10_ (serum triglycerides/serum HDL-C).

### Genotyping

Genomic DNA was extracted from whole blood samples using a standard phenol/chloroform method. Genotyping of the single nucleotide polymorphisms MC4R (rs17782313) was conducted by Polymerase Chain Reaction (PCR) with subsequent restriction fragment length polymorphism assay. PCR amplification of rs17782313 was carried out using designed following primers: forward, 5′ AAGTTCTACCTACCATGTTCTTGG3′ and reverse, 5′ TTCCCCCTGAAGCTTTTCTTGTCATTTTGAT 3′ (Macro-gene, Korea). The volume of each PCR reaction was 20 μl containing 200 ng of DNA, 0.5 μmol of each primer, 10 μl of Taq DNA Polymerase 2 × MasterMix (Ampliqon, Germany). PCR cycles was optimized with an initial denaturation at 95 °C for 2 min, denaturation at 95 °C for 30 s (35 cycles), annealing at 58 °C for 30 s and 30 s of extension at 72 °C. An additional extension occurred at 72 °C for 5 min. PCR products were digested using BclI (Fermentas, Germany) enzyme digestion. Digestion solution (7 μl of PCR product, 0.5 μl of BclI 10 U/μl and 2 μl of 10× restriction G-buffer) was incubated at 56 °C over-night. After that, electrophoresis of the digested PCR products was carried out on 2% agarose gel. To visualize DNA fragments, gels were stained with green viewer (Pars Tous, Iran) on a Gel Doc-system (U.V.P Company, Cambridge, UK). T allele was appeared as fragments with length of 30 and 107 bp. C allele was also considered as a 137 bp fragment.

### Statistical analysis

Normal distribution of data was checked by descriptive measures such as coefficients of skewness and kurtosis, mean and standard deviation [40]. The data were presented as frequency (%) for categorical variables and as mean ± SD for normally distributed continuous variables. Data that were not normally distributed were expressed as median (25th and 75th percentile). Continuous variables including glucose, HOMA-IR, insulin, α-MSH and AgRP that were not normally distributed were log-transformed for analyses that required normal data. The relationships between general characteristics of participants and rs17782313 genotypes were analyzed using the analysis of variance (ANOVA) and chi-square tests. Sex-stratified analysis of covariance (ANCOVA) was used to compare clinical and biochemical parameters between three groups of genotypes by adjusting for the confounding effects of age, physical activity and WC. The association between diet quality indices and rs17782313 genotypes were evaluated by multivariate multinomial logistic regression in different models. Sex-stratified analysis of covariance (ANCOVA) multivariate interaction models using the General Linear Model procedures was used to test the interactions between MC4R polymorphism (rs17782313) and diet quality indices in relation to cardio-metabolic risk factors. After adjusting for confounding factors (age, physical activity and WC), significant interactions were presented as graph to help their illustration. In all interaction analyses, homozygous wild-type group was considered as reference. All statistical analyses were done using Statistical Package for Social Sciences (SPSS, Inc., Chicago, IL, version 21). A *P* value ≤0.05 was considered statistically significant.

## Results

Demographic, genetic and mental characteristics of participants across MC4R rs17782313 genotype are presented in Table 1. There was no significant difference regarding anthropometric and socio-demographic variables and mental health parameters across MC4R rs17782313 genotype. The C-allele of rs17782313 was minor allele and had a frequency of 37% and accordingly T-allele was major allele.

**Table 1:** General characteristics of participants according to MC4R rs17782313 genotypes

	Genotype			
	TT	TC	CC	*P*-value*
Gender				0.157
Men, n (%)	35 (50.7)	23 (33.4)	11 (15.9)	
Women, n (%)	28 (38.9)	28 (38.9)	16 (22.2)	
Age (y)	38.9 (7.8)	38.1 (7.3)	37.2 (7.6)	0.606
WC	108.9 (10.0)	108.8 (9.6)	105.4 (8.2)	0.232
BMI (kg/m^2)^	34.5 (3.6)	34.4 (3.2)	34.3 (3.4)	0.958
Physical activity level, n (%)				0.253
Low	29 (40.8)	28 (39.4)	14 (19.7)	
Moderate	12 (36.4)	15 (45.5)	6 (18.2)	
High	22 (59.5)	8 (21.6)	7 (18.9)	
Marital status, n (%)				0.776
Married	53 (43.8)	45 (37.2)	23 (19.0)	
Single	10 (50.0)	6 (30.0)	4 (20.0)	
SES score	10.3 (2.3)	9.8 (2.5)	9.2 (2.6)	0.107
Depression, n (%)				0.703
Normal	30 (47.6)	21 (33.3)	12 (19.1)	
Mild	8 (36.4)	12 (54.5)	2 (9.1)	
Moderate	16 (47.0)	9 (26.5)	9 (26.5)	
Severe	5 (50.0)	2 (20.0)	3 (30.0)	
Extremely severe	4 (33.3)	7 (58.4)	1 (8.3)	
Anxiety, n (%)				0.417
Normal	23 (45.1)	20 (39.2)	8 (15.7)	
Mild	6 (46.2)	5 (38.4)	2 (15.4)	
Moderate	17 (47.2)	13 (36.1)	6 (16.7)	
Severe	7 (43.7)	4 (25.0)	5 (31.3)	
Extremely severe	10 (40.0)	9 (36.0)	6 (24.0)	
Stress, n (%)				0.343
Normal	25 (43.9)	22 (38.6)	10 (17.5)	
Mild	9 (36.0)	8 (32.0)	8 (32.0)	
Moderate	16 (45.7)	13 (37.1)	6 (17.2)	
Severe	9 (50.0)	6 (33.3)	3 (16.7)	
Extremely severe	4 (66.7)	2 (33.3)	0 (0.0)	
Appetite	33.1 (10.1)	33.2 (8.4)	34.4 (7.5)	0.824

*Analysis of variance for continuous variables and χ^2^ test for categorical variables. Data are Mean ± SD

*Abbreviations*: *BMI* body mass index, *WC* waist circumference, *SES* socio-economic status

**Fig. 1:**
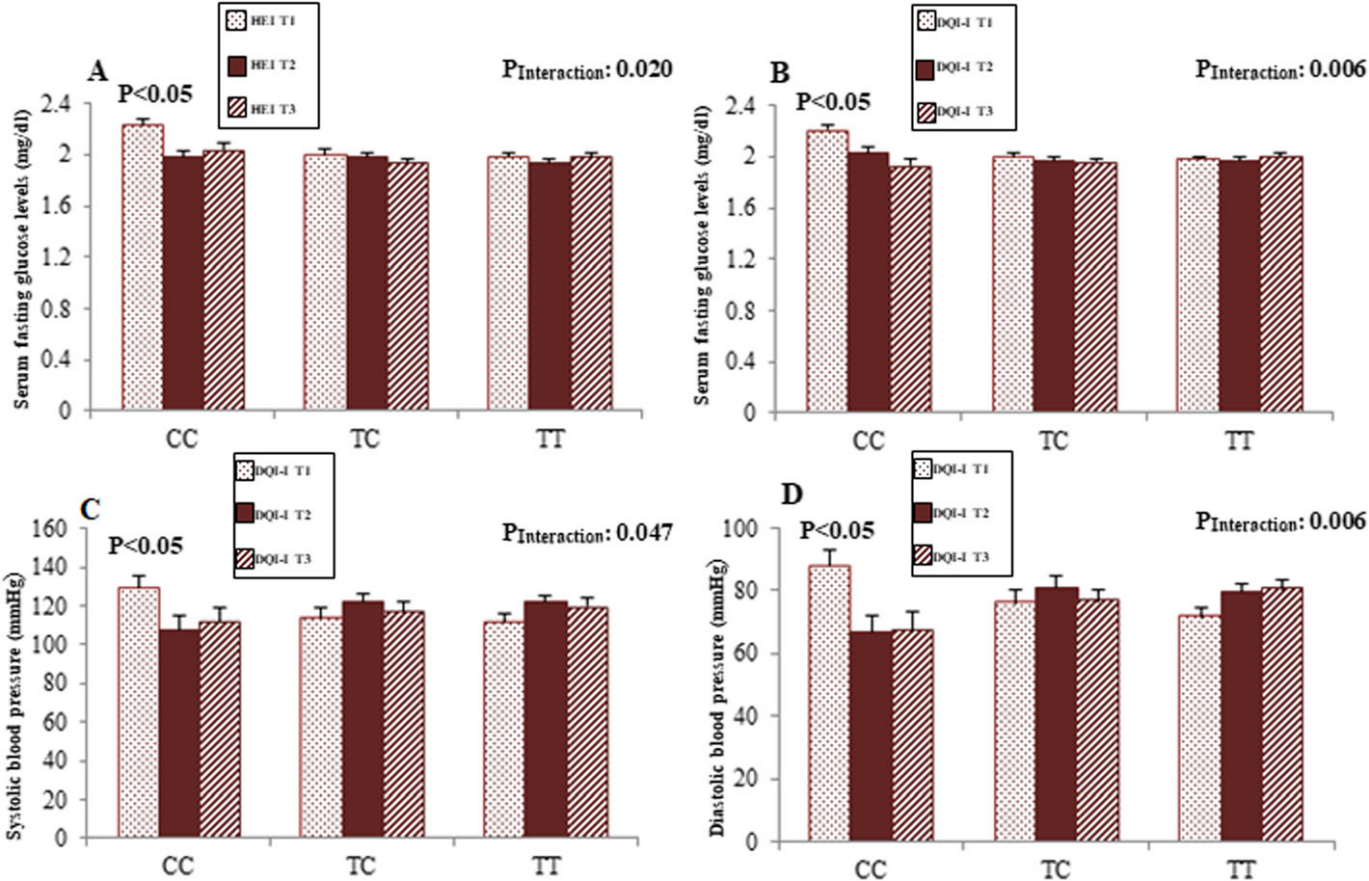
Interaction between the MC4R rs17782313 polymorphisms and HEI-2015 on serum concentrations of glucose among men (**a**), interaction between the rs17782313 SNP and DQI-I on serum concentrations of glucose (**b**), Systolic blood pressure (**c**) and Diastolic blood pressure (**d**) among men. *p*.values of interactions were adjusted for age, WC and physical activity. The bars indicate mean. Error bars: SE of means.

**Fig. 2:**
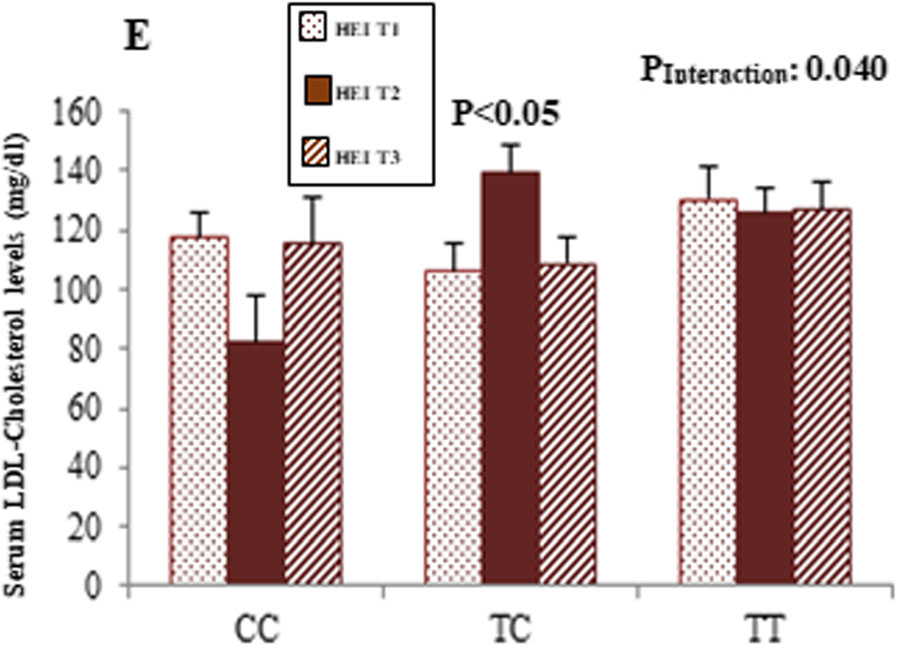
Interaction between the rs17782313 SNP and HEI-2015 on serum concentrations of LDL-Cholesterol among women (**e**). *p*.values of interactions were adjusted for age, WC and physical activity. The bars indicate mean. Error bars: SE of means.

Table 2 shows sex-stratified analysis for the association between clinical and biochemical parameters and MC4R rs17782313 polymorphisms. In men, being in the CC genotype group was associated with higher serum glucose concentrations (*P* = 0.016). This association remained significant even after adjustment for age, WC and physical activity (*P* = 0.012). Moreover, men in CC genotype group showed lower plasma AgRP level than those in TT and CT genotype groups, either in the crude (*P* = 0.025) or the adjusted (*P* = 0.046). However, no statistical significant difference was seen regarding biochemical parameters across MC4R rs17782313 genotypes among women. Sex-stratified analysis for the association between dietary indices and rs17782313 genotypes are presented in Table 3. In female subjects, there was an inverse association between HEI score and odds of having CC genotype when compared to homozygote group with major allele (OR, 0.87; 95% CI, 0.78–0.96). This association remained consistently significant even after adjusting for various potential confounders in multi-adjusted models (OR, 0.88; 95% CI, 0.78–0.98). There was no association between dietary quality indices (HEI and DQI-I) and rs17782313 genotypes; neither in crude nor in multi-adjusted models in men. Sex-stratified interaction analysis was performed to assess modification effects of dietary quality indices (HEI and DQI-I) on the association between MC4R variant and lipid profile and glycemic indices. Significant interactions among men and women are illustrated in Figs. 1 and 2, respectively. Among women, no gene-diet interaction was found except for HEI and rs17782313 in associations with LDL-C levels (P-interaction = 0.044). This interaction remained statistically significant after adjusting for age, physical activity and WC (P _Interaction_ = 0.040). In particular, the CT-genotype carriers who placed in the second tertile of HEI had the highest LDL-C level (*P* < 0.05). On the other hand, we found a gene–diet interaction between rs17782313 genotypes and both HEI (P _Interaction_ = 0.020) and DQI-I (P _Interaction_ = 0.006) for glucose level in male subjects. Such that subjects with the homozygous minor allele genotype were characterized by higher serum glucose concentrations when had lowest adhere to HEI and DQI-I. Furthermore, a significant interaction was detected between DQI-I and rs17782313 for SBP (P _Interaction_ = 0.047) and also DBP (P _Interaction_ = 0.006) after controlling for potential confounder. The highest mean of SBP and DBP was observed in CC genotype group in the first tertile of DQI-I, compared to other tertiles of this index (*P* < 0.05). No significant interaction was found between MC4R rs17782313 polymorphisms and diet quality indices (HEI and DQI-I) on hypothalamic hormones in both men and women.

**Table 2:** Comparison of clinical and laboratory parameters of participants according to MC4R rs17782313 genotypes

	Women					Men				
	TT	TC	CC	P*	P**	TT	TC	CC	P*	P**
LDL-C, (mg/dl)	126.9 (29.60)	118.56 (31.06)	108.91 (28.09)	0.161	0.153	120.46 (26.31)	118.30 (30.01)	108.58 (28.41)	0.471	0.533
HDL, (mg/dl)	48.46 (10.27)	46.37 (10.19)	49.44 (6.46)	0.549	0.720	41.11 (6.64)	43.35 (9.22)	44.45 (9.61)	0.388	0.421
Cholesterol, (mg/dl)	197.71 (33.88)	185.74 (35.45)	177.75 (28.62)	0.146	0.120	188.4 (28.39)	187.78 (31.20)	184.09 (43.36)	0.918	0.953
TG, (mg/dl)	111.75 (46.33)	104.07 (41.84)	97 (35.23)	0.530	0.430	122 (80, 169)	121 (90, 164)	123 (90, 184)	0.931	0.873
AIP	−0.0226 (0.240)	−0.032 (0.230)	−0.087 (0.175)	0.631	0.608	0.11(0.23)	0.09 (0.25)	0.12 (0.29)	0.907	0.866
Glucose, (mg/dl)	92.00 (87.25, 97.00)	91 (81, 101)	90.00 (85.25, 94.00)	0.834	0.675	93 (87, 102)	90 (83, 97)	100 (87, 159)	**0.016**	**0.012**
Insulin, U/mL	13.55 (9.28, 24.10)	19.60 (9.70, 27.30)	13.75 (9.00, 16.23)	0.306	0.278	11.40 (8.90, 17.20)	10.90 (8.40, 19.50)	11.00 (9.30, 24.80)	0.507	0.439
HOMA-IR^a^	3.17 (2.00, 5.56)	4.82 (2.01, 6.58)	3.15 (2.09, 3.56)	0.409	0.395	2.70 (1.78, 4.13)	2.61 (1.87, 5.04)	4.16 (2.30, 6.70)	0.077	0.061
QUICKI	0.32 (0.03)	0.32 (0.04)	0.33 (0.03)	0.466	0.345	0.33 (0.03)	0.33 (0.03)	0.31 (0.03)	0.126	0.104
SBP (mmHg)	111.86(14.48)	116.64 (16.85)	111.19 (14.40)	0.404	0.716	117.71 (12.45)	118.70 (15.83)	115.4 (13.68)	0.816	0.966
DBP (mmHg)	76.5 (10.11)	78.82 (14.38)	75.5 (11.68)	0.644	0.810	77 (10.79)	78.26 (10.93)	74.09 (11.36)	0.584	0.664
α-MSH (ng/L)	2.16 (2.12–2.20)	2.26 (2.17–2.35)	2.26 (2.11–2.41)	0.153	0.259	2.29 (2.20–2.37)	2.39 (2.25–2.52)	2.18 (2.09–2.27)	0.105	0.175
AgRP (pg/ml)	1.33 (1.29–1.37)	1.43 (1.36–1.51)	1.41 (1.29–1.53)	0.082	0.176	1.49 (1.42–1.57)	1.57 (1.46–1.68)	1.33 (1.25–1.41)	**0.025**	**0.046**

Data are presented as, mean (SD) or geometric mean (95% CI). Analysis of variance for continuous variables and χ^2^ test for categorical variables. *P values based on One-Way ANOVA, **P values adjusted for age, physical activity and WC *Abbreviations*: *HOMA-IR* homeostasis model assessment of insulin resistance, *LDL-C* low density lipoprotein cholesterol, *HDL* high-density lipoprotein, *SBP* systolic blood pressure, *DBP* diastolic blood pressure, *TG* triglyceride, *QUICKI* quantitative insulin sensitivity check index, *AgRP* agouti-related protein, *α-MSH* alpha melanocyte stimulating hormone, *AIP* athrogenic indx of plasma. Bold values provide significant treshold as *P <0.05*

**Table 3:** Odd’s ratio (OR) and confidence interval (CI) for the association between dietary quality indices and MC4R rs17782313 genotypes

	TT	Women	CC	TT	Men	CC
		TC			TC	
HEI. Total score						
Crude	1(Ref.)	0.99 (0.93–1.06)	**0.87 (0.78–0.9**6)	1(Ref.)	1.05 (0.97–1.13)	1.02 (0.93–1.11)
Model 1*	1(Ref.)	1.00 (0.93–1.08)	**0.86 (0.78–0.98)**	1(Ref.)	1.06 (0.98–1.15)	1.02 (0.93–1.13)
Model 2**	1(Ref.)	1.01 (0.93–1.09)	**0.88 (0.78–0.98)**	1(Ref.)	1.06 (0.98–1.14)	1.02 (0.93–1.13)
DQI.I. Total score						
Crude	1(Ref.)	1.00 (0.94–1.06)	0.94 (0.87–1.01)	1(Ref.)	1.05 (0.98–1.13)	0.97 (0.89–1.07)
Model 1	1(Ref.)	0.99 (0.93–1.06)	0.94 (0.87–1.02)	1(Ref.)	1.06 (0.99–1.15)	0.98 (0.89–1.08)
Model 2	1(Ref.)	1.00 (0.93–1.07)	0.94 (0.87–1.02)	1(Ref.)	1.06 (0.99–1.14)	0.98 (0.89–1.08)

The multivariate multinomial logistic regression was used for estimation of ORs and confidence interval (CI). *Adjusted for age, physical activity and WC. ** Additionally adjusted for stress. Bold values provide significant treshold as *P < 0.05*

## Discussion

To our knowledge, our study is the first to report the interaction between MC4R rs17782313 polymorphisms and diet quality indices on cardio-metabolic traits. In the present study, we found that adherence to the HEI modified the effects of MC4R rs17782313 polymorphisms (rs17782313) on levels of LDL-C in women and glucose in men. Among female group, rare allele heterozygotes of rs17782313 had highest mean of LDL-C concentration when placed in second tertile of HEI. Furthermore, in male group, a gene-diet interaction was detected between DQI-I and MC4R rs17782313 on glucose level, SBP and DBP. Statistical adjustment for age, physical activity and WC did not change the statistical significance of these gene–diet interactions in both men and women. Among male homozygous carriers of the risk allele, those with lowest adherence to HEI and DQI-I had highest glucose concentrations. Another novel finding of our study was the inverse association between adherence to the HEI and chance of having CC genotype among female group. In addition, minor homozygote carriers of rs17782313 had lower adjusted means of AgRP levels compared with other genotypes (TT and TC).

Various range of minor allele frequency of rs17782313 polymorphism has been reported in different populations from 14% in Asians to 28% in Europeans. However, current study showed a frequency of 37% in Iranian population. Despite interactions between MC4R genes and dietary factors on obesity and other metabolic traits have been investigated in several studies [25, 41], significant results have been reported only in limited number of investigations [42]. In this regard, Ortega-Azorín et al. found that adherence to the Mediterranean dietary pattern (MedDiet) could modify genetic susceptibility to diabetes [43]. In that study, CC-genotype carriers of the MC4R rs17782313 with low adherence to the MedDiet had a higher risk of Type 2 diabetes. To the best of our knowledge, specific interaction of variant rs17782313 gene with dietary intakes on cardio-metabolic risk factors is unknown. In the present research, among males with susceptible genotypes, individuals with poor adherence to HEI and DQI-I exhibited a higher SBP, DBP and glucose concentration. Our finding in this regard are supported by animal studies in which homozygous mutated mice exposed with high fat diet, exhibited hyperphagia and weight gain compared with wild-type mice [44]. Moreover, finding of a cohort study confirmed that improving adherence to diet quality represented by higher scores of alternate healthy eating index (AHEI)-2010 and dietary approach to stop hypertension (DASH) could attenuate the genetic association with weight gain particularly in subjects at high genetic risk for obesity [45]. On the other hand, our data indicated among female group, CT-genotype carriers who placed in the second tertile of HEI had the highest LDL-C concentrations. Gender-dependent differences regard to the association between rs17782313 and obesity-related traits have previously recognized [46]. The reason for this discrepancy between men and women is unclear but might be related to difference in hormonal status and regional depots of adipose tissue [47]. There is increasing evidence that polymorphism of rs17782313 (minor C allele) is related to some of cardio-metabolic risk factors such as hypertriglyceridemia [48], low HDL-cholesterol [49] and high LDL and total cholesterol [24]. Likewise, we found the homozygous males for the minor allele (C) had higher adjusted means of serum glucose levels compared with the other genotypes (TT and TC). Our finding in this regard was in line with previous observational studies that SNP rs17782313 was significantly associated with insulin resistance [50] and enhanced risk of diabetes [51]. In the present study, HEI score was negatively associated with polymorphism of rs17782313 (CC genotype) among female group. Similarly, there is evidence that indicate rs17782313 SNP of MC4R gene influence on dietary intake parameters [51]. For example, Qi et al. in a large cohort study indicated that rs17782313 variant was associated with higher intakes of total energy and dietary fat [51]. Nevertheless, the evidence from other human investigations in this regard is inconsistent [52]. In the current study, adherence to healthy dietary patterns, which consider the effects of whole diet instead of focusing on single foods and macro- and micronutrients in isolation, were assessed by diet quality scores. Even though the precise mechanisms behind these observed interactions remain unknown, there is considerable evidence that these diet quality indices (HEI and DQI-I) protects against major diet-dependent chronic diseases [53, 54]. Hence, it is not surprising that lower adherence to these diet quality indices can promote effects of greater genetic susceptibility to cardio-metabolic risk factors. The favorable effects of these indices can be attributed to healthy choice such as vegetables, fruits, whole grains, sea food and plant proteins and more emphasize on polyunsaturated-to saturated fat ratio [10, 54]. In this regard, emerging researches demonstrated the inverse associations of fruit, vegetables and diet-related chronic diseases [55].

Although not much is known about the association between α-MSH, AgRP and MC4R polymorphisms, present investigation showed that homozygous male for the minor allele (C) had lower levels of AgRP than other genotypes. Previous human studies that have investigated plasma AgRP and α-MSH concentration have reported equivocal results [56–58]. However, most of these studies have shown that both plasma α-MSH and AgRP levels increase in obese adult subjects [56, 57]. Evidence had indicated that any alteration in expression of these orexigenic and anorexigenic peptides (α-MSH and AgRP) may play a potential role in energy homeostasis, food intake and development of obesity [59].

Several limitations of the current study should also be addressed; firstly, due to the cross-sectional nature of this study, causality cannot be inferred. Nevertheless, these results can provide hypothesis for prospective studies to evaluate and confirm true causal relation. Secondly, since small sample size may limit statistical power, the results of present study with relatively small sample size should be interpreted with caution until replicated in large longitudinal studies. Thirdly, under-reporting of dietary intakes that are commonly observed in obese individuals, may lead to potential bias and null results [60]. However, subjects with extreme dietary intake values were not included in the analysis. On the other hand, current study as the same as other observational studies is prone to residual confounding due to unknown or unmeasured confounders [61]. Fourthly, we did not have information regarding other modifiable factors such as meal and snacking patterns and dietary habits that may be effective on observed associations. Lastly, this research was carried out in Tabriz, one of the major cities in Iran with a unique culture and dietary intake, so results may not be generalizable to all Iranians and it need to be replicated in other populations. Despite limitations discussed above, this is the first attempt to study the interaction between MC4R rs17782313 polymorphisms and diet quality indices on cardio-metabolic traits, according to our knowledge. Identification of these gene-diet interactions could be crucial in planning appropriate personalized nutritional advice for the prevention and management of obesity and its-related consequences. Another major strengths of present study was use of a reliable [29] and validated [28] FFQ to collect dietary information.

## Conclusions

In conclusion, the finding of present study showed for the first time a statistically significant gene-diet interaction of the MC4R rs17782313 with adherence to the dietary quality indices (HEI and DQI-I) on some of cardio-metabolic risk factors in both genders. In sum, in male group, when adherence to these indices was low, the obesity risk allele (CC) was associated with higher fasting glucose level, SBP and DBP. On the other hand, in female group, medium adherence to HEI by rare allele heterozygotes of rs17782313 increased the risk of having higher LDL-C level. The results of our study should be confirmed in large prospective studies.

## Data Availability

All of the data are available with reasonable request from the corresponding author.

## Abbreviations

AGRP: Agouti-related protein
AHEI: Alternate healthy eating index
AIP: Atherogenic index of plasma
ANCOVA: Analysis of covariance
ANOVA: Analysis of variance
BMI: Body mass index
DASH: Dietary approaches to stop hypertension
DASS-21: Depression, anxiety and stress scale 21
DBP: Diastolic blood pressure
DGA: Dietary guidelines for Americans
DQI-I: Diet quality index-international
ELISA: Enzyme-linked immunosorbent assay
FCT: Food composition table
FFQ: Food frequency questionnaire
GWAS: Genome-wide association studies
HDL-C: High-density lipoprotein cholesterol
HEI-2015: Healthy eating Index-2015
HOMA-IR: Homeostasis model assessment-insulin resistance
LDL-C: Low-density lipoprotein-cholesterol
MC4R: Melanocortin-4 receptor
MedDiet: Mediterranean dietary pattern
NCDs: Non-communicable diseases
PCR-RFLP: Polymerase chain reaction restriction fragment length polymorphism
QUICKI: Quantitative insulin sensitivity check index
SBP: Systolic blood pressure
SES: Socioeconomic status
SNP: Single nucleotide polymorphism
SPSS: Statistical package for social sciences
TG: Triglyceride
VAS: Visual analogue scale
WC: Waist circumference
WHO: World Health Organization
α-MSH: Alpha-melanocyte stimulating hormone

## References

[1] Jafari-Adli S, Jouyandeh Z, Qorbani M, Soroush A, Larijani B, Hasani-Ranjbar S (2014). Prevalence of obesity and overweight in adults and children in Iran; a systematic review. J Diabetes Metab Disord.

[2] Organization WH (2000). Obesity: preventing and managing the global epidemic: World Health Organization.

[3] Rahmani A, Sayehmiri K, Asadollahi K, Sarokhani D, Islami F, Sarokhani M (2015). Investigation of the prevalence of obesity in Iran: a systematic review and meta-analysis study. Acta Medica Iranica.

[4] Dixon JB (2010). The effect of obesity on health outcomes. Mol Cell Endocrinol.

[5] Reilly J, Methven E, McDowell Z (2003). Health consequences of obesity arch dis child.

[6] Hruby A, Manson JE, Qi L, Malik VS, Rimm EB, Sun Q (2016). Determinants and consequences of obesity. Am J Public Health.

[7] Bälter K, Möller E, Fondell E (2012). The effect of dietary guidelines on cancer risk and mortality. Curr Opin Oncol.

[8] Hu FB (2002). Dietary pattern analysis: a new direction in nutritional epidemiology. Curr Opin Lipidol.

[9] Asghari G, Mirmiran P, Yuzbashian E, Azizi F (2017). A systematic review of diet quality indices in relation to obesity. Br J Nutr.

[10] Kim S, Haines PS, Siega-Riz AM, Popkin BM (2003). The diet quality index-international (DQI-I) provides an effective tool for cross-national comparison of diet quality as illustrated by China and the United States. J Nutr.

[11] Asghari G, Mirmiran P, Rashidkhani B, Asghari-Jafarabadi M, Mehran M, Azizi F (2012). The association between diet quality indices and obesity: Tehran lipid and glucose study. Arch Iran Med.

[12] Gregory CO, McCullough ML, Ramirez-Zea M, Stein AD (2009). Diet scores and cardio-metabolic risk factors among Guatemalan young adults. Br J Nutr.

[13] Lassale C, Fezeu L, Andreeva VA, Hercberg S, Kengne AP, Czernichow S (2012). Association between dietary scores and 13-year weight change and obesity risk in a French prospective cohort. Int J Obes.

[14] Guenther PM, Casavale KO, Reedy J, Kirkpatrick SI, Hiza HA, Kuczynski KJ (2013). Update of the healthy eating index: HEI-2010. J Acad Nutr Diet.

[15] Krebs-Smith SM, Pannucci TE, Subar AF, Kirkpatrick SI, Lerman JL, Tooze JA (2018). Update of the healthy eating index: HEI-2015. J Acad Nutr Diet.

[16] Schwingshackl L, Bogensberger B, Hoffmann G (2018). Diet quality as assessed by the healthy eating index, alternate healthy eating index, dietary approaches to stop hypertension score, and health outcomes: an updated systematic review and meta-analysis of cohort studies. J Acad Nutr Diet.

[17] Cone RD (2005). Anatomy and regulation of the central melanocortin system. Nat Neurosci.

[18] Nickolls Sarah A., Cismowski Mary I., Wang Xiaochuan, Wolff Meira, Conlon Paul J., Maki Richard A. (2002). Molecular Determinants of Melanocortin 4 Receptor Ligand Binding and MC4/MC3 Receptor Selectivity. Journal of Pharmacology and Experimental Therapeutics.

[19] Srisai D, Gillum MP, Panaro BL, Zhang XM, Kotchabhakdi N, Shulman GI (2011). Characterization of the hyperphagic response to dietary fat in the MC4R knockout mouse. Endocrinology.

[20] Loos RJ, Lindgren CM, Li S, Wheeler E, Zhao JH, Prokopenko I (2008). Common variants near MC4R are associated with fat mass, weight and risk of obesity. Nat Genet.

[21] Hardy R, Wills AK, Wong A, Elks CE, Wareham NJ, Loos RJ (2010). Life course variations in the associations between FTO and MC4R gene variants and body size. Hum Mol Genet.

[22] Haupt A, Thamer C, Heni M, Tschritter O, Machann J, Schick F (2009). Impact of variation near MC4R on whole-body fat distribution, liver fat, and weight loss. Obesity (Silver Spring, Md).

[23] Grant SFA, Bradfield JP, Zhang H, Wang K, Kim CE, Annaiah K (2009). Investigation of the locus near MC4R with childhood obesity in Americans of European and African ancestry. Obesity (Silver Spring, Md).

[24] Tao L, Zhang Z, Chen Z, Zhou D, Li W, Kan M (2012). A common variant near the melanocortin 4 receptor is associated with low-density lipoprotein cholesterol and total cholesterol in the Chinese Han population. Mol Biol Rep.

[25] Taylor AE, Sandeep MN, Janipalli CS, Giambartolomei C, Evans DM, Kranthi Kumar MV (2011). Associations of FTO and MC4R variants with obesity traits in Indians and the role of rural/urban environment as a possible effect modifier. J Obes.

[26] Casazza K, Dulin-Keita A, Gower BA, Fernandez J (2009). Differential influence of diet and physical activity on components of metabolic syndrome in a multiethnic sample of children. J Am Diet Assoc.

[27] Koochakpoor G, Hosseini-Esfahani F, Daneshpour MS, Hosseini SA, Mirmiran P (2016). Effect of interactions of polymorphisms in the Melanocortin-4 receptor gene with dietary factors on the risk of obesity and type 2 diabetes: a systematic review. Diabet Med.

[28] Mirmiran P, Esfahani FH, Mehrabi Y, Hedayati M, Azizi F (2010). Reliability and relative validity of an FFQ for nutrients in the Tehran lipid and glucose study. Public Health Nutr.

[29] Esfahani FH, Asghari G, Mirmiran P, Azizi F (2010). Reproducibility and relative validity of food group intake in a food frequency questionnaire developed for the Tehran lipid and glucose study. J Epidemiol.

[30] Ghaffarpour M, Houshiar-Rad M, Kianfar H (1999). The manual for household measures, cooking yields factors and edible portion of foods.

[31] Gaeini Z, Bahadoran Z, Mirmiran P, Djazayery A (2019). The association between dietary fat pattern and the risk of type 2 diabetes. Prev Nutr Food Sci.

[32] Asghari G, Yuzbashian E, Mirmiran P, Azizi F (2017). The association between dietary approaches to stop hypertension and incidence of chronic kidney disease in adults: the Tehran Lipid and glucose study. Nephrol Dial Transplant.

[33] Booth M (2000). Assessment of physical activity: an international perspective. Res Q Exerc Sport.

[34] Samani S, Jokar B (2008). Validity and reliability short-form version of the Depression, Anxiety and Stress.

[35] Sahebi A, Asghari MJ, Salari RS (2005). Validation of depression anxiety and stress scale (DASS-21) for an Iranian population. Scientific J Manag Syst.

[36] Flint A, Raben A, Blundell JE, Astrup A (2000). Reproducibility, power and validity of visual analogue scales in assessment of appetite sensations in single test meal studies. Int J Obes Relat Metab Disord.

[37] Friedewald WT, Levy RI, Fredrickson DS (1972). Estimation of the concentration of low-density lipoprotein cholesterol in plasma, without use of the preparative ultracentrifuge. Clin Chem.

[38] Matthews DR, Hosker JP, Rudenski AS, Naylor BA, Treacher DF, Turner RC (1985). Homeostasis model assessment: insulin resistance and beta-cell function from fasting plasma glucose and insulin concentrations in man. Diabetologia.

[39] Katz A, Nambi SS, Mather K, Baron AD, Follmann DA, Sullivan G (2000). Quantitative insulin sensitivity check index: a simple, accurate method for assessing insulin sensitivity in humans. J Clin Endocrinol Metab.

[40] Das K (2016). A brief review of tests for normality.

[41] Holzapfel C, Grallert H, Huth C, Wahl S, Fischer B, Doring A (2010). Genes and lifestyle factors in obesity: results from 12,462 subjects from MONICA/KORA. Int J Obes (2005).

[42] Robitaille J, Perusse L, Bouchard C, Vohl MC (2007). Genes, fat intake, and cardiovascular disease risk factors in the Quebec Family Study. Obesity (Silver Spring, Md).

[43] Ortega-Azorin C, Sorli JV, Asensio EM, Coltell O, Martinez-Gonzalez MA, Salas-Salvado J (2012). Associations of the FTO rs9939609 and the MC4R rs17782313 polymorphisms with type 2 diabetes are modulated by diet, being higher when adherence to the Mediterranean diet pattern is low. Cardiovasc Diabetol.

[44] Butler AA, Cone RD (2003). Knockout studies defining different roles for melanocortin receptors in energy homeostasis. Ann N Y Acad Sci.

[45] Wang T, Heianza Y, Sun D, Huang T, Ma W, Rimm EB (2018). Improving adherence to healthy dietary patterns, genetic risk, and long term weight gain: gene-diet interaction analysis in two prospective cohort studies. BMJ (Clinical research ed).

[46] Bjornland T, Langaas M, Grill V, Mostad IL (2017). Assessing gene-environment interaction effects of FTO, MC4R and lifestyle factors on obesity using an extreme phenotype sampling design: results from the HUNT study. PLoS One.

[47] Pinnick KE, Nicholson G, Manolopoulos KN, McQuaid SE, Valet P, Frayn KN (2014). Distinct developmental profile of lower-body adipose tissue defines resistance against obesity-associated metabolic complications. Diabetes.

[48] Katsuura-Kamano S, Uemura H, Arisawa K, Yamaguchi M, Hamajima N, Wakai K (2014). A polymorphism near MC4R gene (rs17782313) is associated with serum triglyceride levels in the general Japanese population: the J-MICC study. Endocrine.

[49] Kring SI, Holst C, Toubro S, Astrup A, Hansen T, Pedersen O (2010). Common variants near MC4R in relation to body fat, body fat distribution, metabolic traits and energy expenditure. Int J Obes (2005).

[50] Chambers JC, Elliott P, Zabaneh D, Zhang W, Li Y, Froguel P (2008). Common genetic variation near MC4R is associated with waist circumference and insulin resistance. Nat Genet.

[51] Qi L, Kraft P, Hunter DJ, Hu FB (2008). The common obesity variant near MC4R gene is associated with higher intakes of total energy and dietary fat, weight change and diabetes risk in women. Hum Mol Genet.

[52] Corella D, Ortega-Azorin C, Sorli JV, Covas MI, Carrasco P, Salas-Salvado J (2012). Statistical and biological gene-lifestyle interactions of MC4R and FTO with diet and physical activity on obesity: new effects on alcohol consumption. PLoS One.

[53] Cheung LTF, Chan RSM, Ko GTC, Lau ESH, Chow FCC, Kong APS (2018). Diet quality is inversely associated with obesity in Chinese adults with type 2 diabetes. Nutr J.

[54] Onvani S, Haghighatdoost F, Surkan PJ, Larijani B, Azadbakht L (2017). Adherence to the healthy eating index and alternative healthy eating index dietary patterns and mortality from all causes, cardiovascular disease and cancer: a meta-analysis of observational studies. J Hum Nutr Diet.

[55] Esmaillzadeh A, Kimiagar M, Mehrabi Y, Azadbakht L, Hu FB, Willett WC (2006). Fruit and vegetable intakes, C-reactive protein, and the metabolic syndrome. Am J Clin Nutr.

[56] Hoggard N, Johnstone AM, Faber P, Gibney ER, Elia M, Lobley G (2004). Plasma concentrations of alpha-MSH, AgRP and leptin in lean and obese men and their relationship to differing states of energy balance perturbation. Clin Endocrinol.

[57] Katsuki A, Sumida Y, Gabazza EC, Murashima S, Tanaka T, Furuta M (2001). Plasma levels of agouti-related protein are increased in obese men. J Clin Endocrinol Metab.

[58] Nam SY, Kratzsch J, Kim KW, Kim KR, Lim SK, Marcus C (2001). Cerebrospinal fluid and plasma concentrations of leptin, NPY, and alpha-MSH in obese women and their relationship to negative energy balance. J Clin Endocrinol Metab.

[59] Stutz AM, Morrison CD, Argyropoulos G (2005). The agouti-related protein and its role in energy homeostasis. Peptides.

[60] Fisher JO, Johnson RK, Lindquist C, Birch LL, Goran MI (2000). Influence of body composition on the accuracy of reported energy intake in children. Obes Res.

[61] Norgaard M, Ehrenstein V, Vandenbroucke JP (2017). Confounding in observational studies based on large health care databases: problems and potential solutions - a primer for the clinician. Clin Epidemiol.

